# Supplementary material for: Organo–organic and organo–mineral interfaces in soil at the nanometer scale
Source: Nat Commun. 2020 Nov 30;11:6103. doi: 10.1038/s41467-020-19792-9 (PMC7705750; doi:10.1038/s41467-020-19792-9)
Supplement: Supplementary file 1 — Supplementary Information [file 41467_2020_19792_MOESM1_ESM.pdf]

Supplementary Information for:

Organo-organic and organo-mineral interfaces in soil at the nanometer scale

by

Angela R. Possinger[1,2], Michael J. Zachman[3,4], Akio Enders[1,5], Barnaby D. A. Levin[3], David A. Muller[3,6], Lena F. Kourkoutis[3,6], Johannes Lehmann[1,7,8]*

[1]Soil and Crop Sciences, School of Integrative Plant Science, Cornell University, Ithaca, NY 14853, USA.

[2]Present address: Department of Forest Resources and Environmental Conservation, Virginia Tech, Cheatham Hall, Blacksburg, VA 24060, USA.

[3]School of Applied and Engineering Physics, Cornell University, Ithaca, NY 14853, USA.

[4]Present address: Center for Nanophase Materials Sciences, Oak Ridge National Laboratory, Oak Ridge, TN 37831, USA.

[5]Present address: Department of Biological and Environmental Engineering, Cornell University, Ithaca, NY 14853, USA.

[6]Kavli Institute at Cornell for Nanoscale Science, Cornell University, Ithaca, NY 14853, USA.

[7]Institute for Advanced Study, Technical University of Munich, Garching, Germany.

[8]Cornell Atkinson Center for Sustainability, Ithaca NY 14853, USA.

*Correspondence to: CL273@cornell.edu

Supplementary Tables

Supplementary Table 1. **Sampling location and soil properties.** Soil properties of samples used for cryogenic scanning transmission electron microscopy with electron energy loss spectroscopy (STEM-EELS) preparations. Two approaches to cryo-FIB milling (Supplementary Fig. 8) were completed using similar Andisol samples. Oxalate-extractable iron (Fe), aluminum (Al), total organic carbon (TOC), and soil pH data are from ref. 1. Bulk radiocarbon (^{14}C) fraction modern (Fm) value for organo-organic interface sample from refs. 2-4.

	Organo-mineral Interface	Organo-organic Interface
Sample location	Kohala, HI	Kohala, HI
Elevation (m)	1195	1271
Precipitation (m)	1.784	1.918
Sampling depth (m)	0.7-0.9	0.7-0.9
Oxalate-extractable Fe (g kg^{-1})	11.3	28.7
Oxalate-extractable Al (g kg^{-1})	72.7	106.1
TOC (g kg^{-1})	56.8	150.2
Fraction modern (Fm)	No data	0.28
pH	6.7	4.7

Supplementary Table 2. **Nitrogen and oxidized carbon enrichment at organo-mineral interface.** Calculated ratios of integrated area for carbon (C) (280.0-315.0 eV) and nitrogen (N) (395.0-430.0 eV), and ratio of higher-energy (286.6-289.0 eV) (region x in main text Fig. 2b) to lower-energy (284.0-286.5 eV) (region y) integrated intensity normalized to total C integrated intensity at the organo-mineral interface and adjacent C region in a volcanic soil sample (main text Fig. 2).

Mineral-organic adjacent vs. interface		
Point	C/N*	High/low energy region ratio (x/y)
1	36.9	1.1
2	80.1	1.0
3	79.3	1.0
Average	65.4	1.0
Linescan of organo-mineral interface		
Point	C/N*	High/low energy region ratio (x/y)
A	7.1	1.4
B	10.4	1.5
C	9.9	1.3
D	6.6	1.3
E	6.1	1.4
Average	8.0	1.4
Interface change (%)		
Average	88	33
Minimum	72	16
Maximum	92	53

*The value of calculated ratios does not correspond to sample elemental ratios due to cross-sectional area uncertainty.

Supplementary Table 3**. Nitrogen and alkyl carbon enrichment at organo-organic interface.** Calculated ratios of integrated area for carbon (C) (280.0-315.0 eV), nitrogen (N) (395.0-430.0 eV), and oxygen (O) (530.0-565.0 eV), and ratio of aromatic (284.25-285.75 eV) to alkyl (286.0-287.5 eV) integrated intensity normalized to total C integrated intensity across the interface between two organic phases in a volcanic soil sample (main text Figs 3-5).

Organo-organic interface			
Box	C/N	C/O	Aromatic/alkyl C
1	74.1	25.9	0.75
2	28.6	29.7	0.74
3	12.0	32.6	0.72
4	7.7	59.9	0.68
5	8.2	73.6	0.71
Interface change (%)			
Box 4 vs. 5	6.9	18.6	4.0

*The value of calculated EELS intensity ratio does not correspond to exact sample elemental ratios due to cross-sectional area uncertainty.

Supplementary Table 4. **Electron energy loss spectroscopy parameters.** Summary of cryo-electron energy loss spectroscopy (EELS) data collection parameters. FOV = field of view; F20 = FEI F20 TEM-STEM instrument; Titan = FEI Titan Themis S/TEM instrument. Approximate energy resolution is based on the full-width at half-maximum of the zero-loss peak.

Description	Figures	EELS data type	Instrument	Beam current (nA)	Energy shift (eV)	Energy resolution (eV)	Dose ($e^- Å^{-2}$)
Organo-mineral interface linescan	2	Line scan	F20 (200 kV)	0.010	+2.1	1.1	1.56×10^8 (per point)
Adjacent carbon point 1	2	Point scan	F20 (200 kV)	0.010	NA	1.1	1.56×10^8
Adjacent carbon point 2	2	Point scan	F20 (200 kV)	0.0175	NA	1.1	4.38×10^8
Adjacent carbon point 3	2	Point scan	F20 (200 kV)	0.0175	NA	1.1	5.47×10^8
Organo-organic overview	3	Map	Titan (120 kV)	0.025	NA	1.8	1.76×10^0
Organic patches	3	Map	Titan (120 kV)	0.025	Paired to DualEELS	1.8	3.88×10^1
Organo-organic interface	4-5	EELS map	Titan (120 kV)	0.025	Paired to DualEELS	1.8	1.10×10^4
Damage series A	SI 11	Scan over FOV	Titan (120 kV)	0.16	-2.5	0.5	1.03×10^4
Damage series B	SI 11	Scan over FOV	Titan (120 kV)	0.16	-2.5	0.5	1.99×10^4
Damage series C	SI 11	Scan over FOV	Titan (120 kV)	0.16	-2.5	0.5	2.96×10^4
Damage series D	SI 11	Scan over FOV	Titan (120 kV)	0.16	-2.5	0.5	3.92×10^4
Damage series E	SI 11	Scan over FOV	Titan (120 kV)	0.16	-2.5	0.5	4.82×10^4
Damage series F	SI 11	Scan over FOV	Titan (120 kV)	0.16	-2.5	0.5	5.72×10^4
Damage series G	SI 11	Scan over FOV	Titan (120 kV)	0.16	-2.5	0.5	6.69×10^4

Supplementary Figures

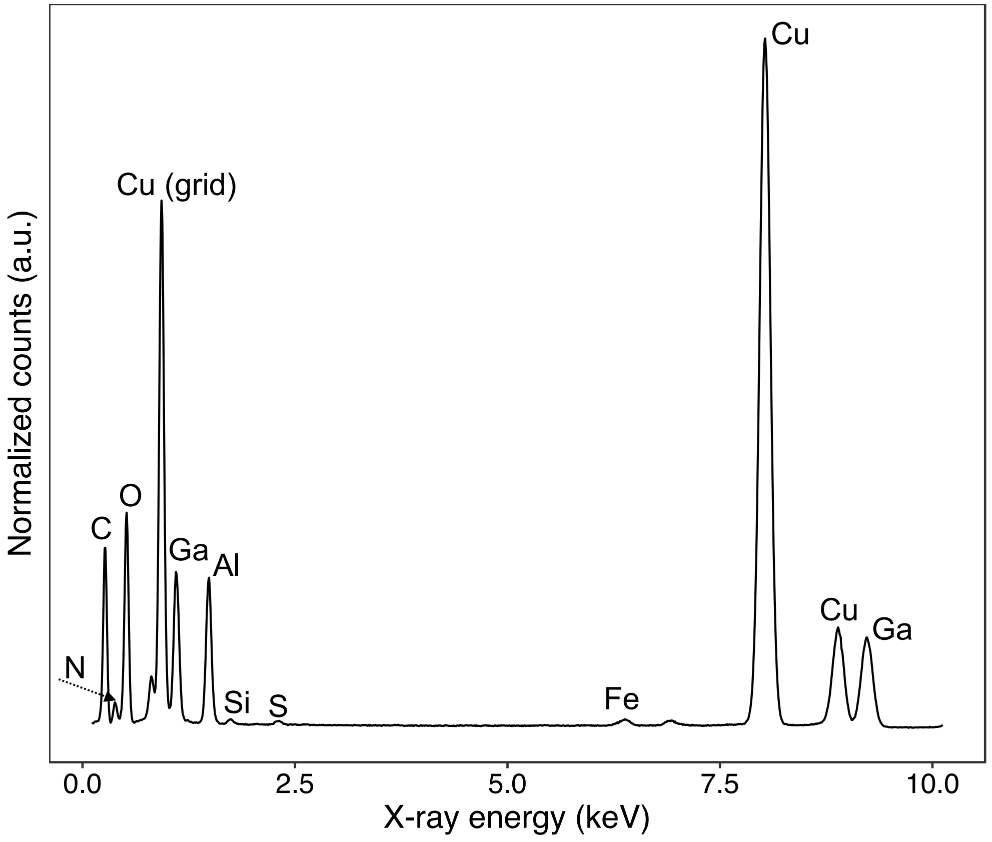

Supplementary Figure 1**. Organo-mineral sample electron dispersive X-ray spectrum**. Scanning transmission electron microscopy-electron dispersive X-ray (STEM-EDX) sum spectrum (spline curve) (0.1-10 keV) for mineral region in proximity to mineral-organic interface in a volcanic soil sample analyzed with STEM-EELS (wedge sample). Elements were assigned to peaks using K-α edge energy positions.

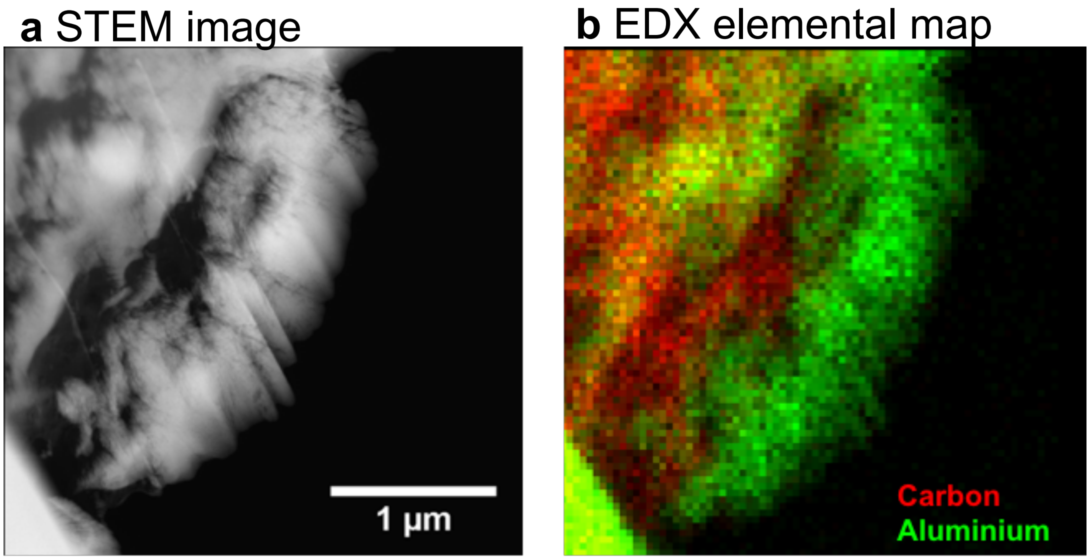

Supplementary Figure 2. **Organo-mineral sample electron dispersive X-ray map. a** High-angle annular dark field (HAADF) scanning transmission electron microscopy (STEM) overview image of volcanic soil sample thin section with organo-mineral interface analyzed by point and linescan EELS. **b** Elemental electron dispersive X-ray (EDX) spectroscopy map showing layered structures visible within the dark field image identified as aluminum, with adjacent regions high in carbon.

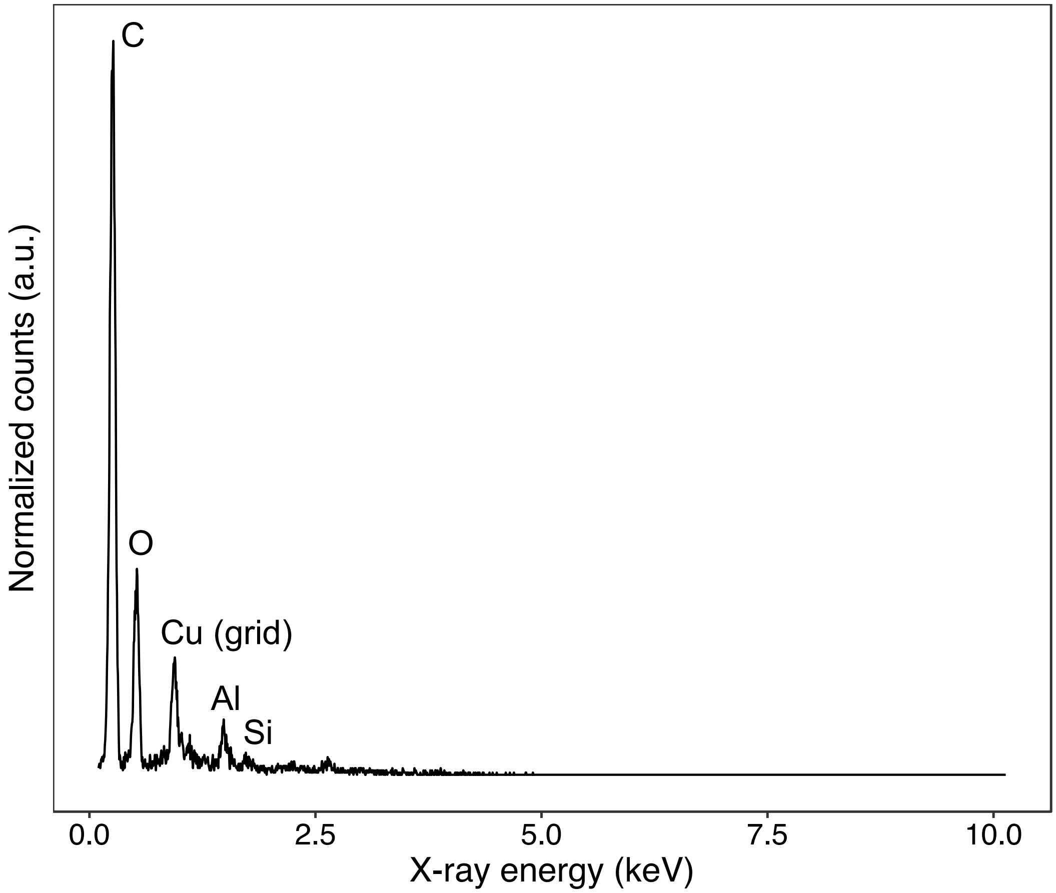

Supplementary Figure 3. **Organo-organic sample electron dispersive X-ray spectrum**. Scanning electron microscopy electron dispersive X-ray (SEM-EDX) spectrum (collected at 5kV) from point scan of volcanic soil sample for subsequent organo-organic interface EELS analysis prior to focused ion beam (FIB) milling and sample prep. EDX spectrum (spline curve) shows low Al and high C content. No features >4 keV were identified in this analysis due to 5 kV collection voltage. Elements were assigned to peaks using K-α edge energy positions.

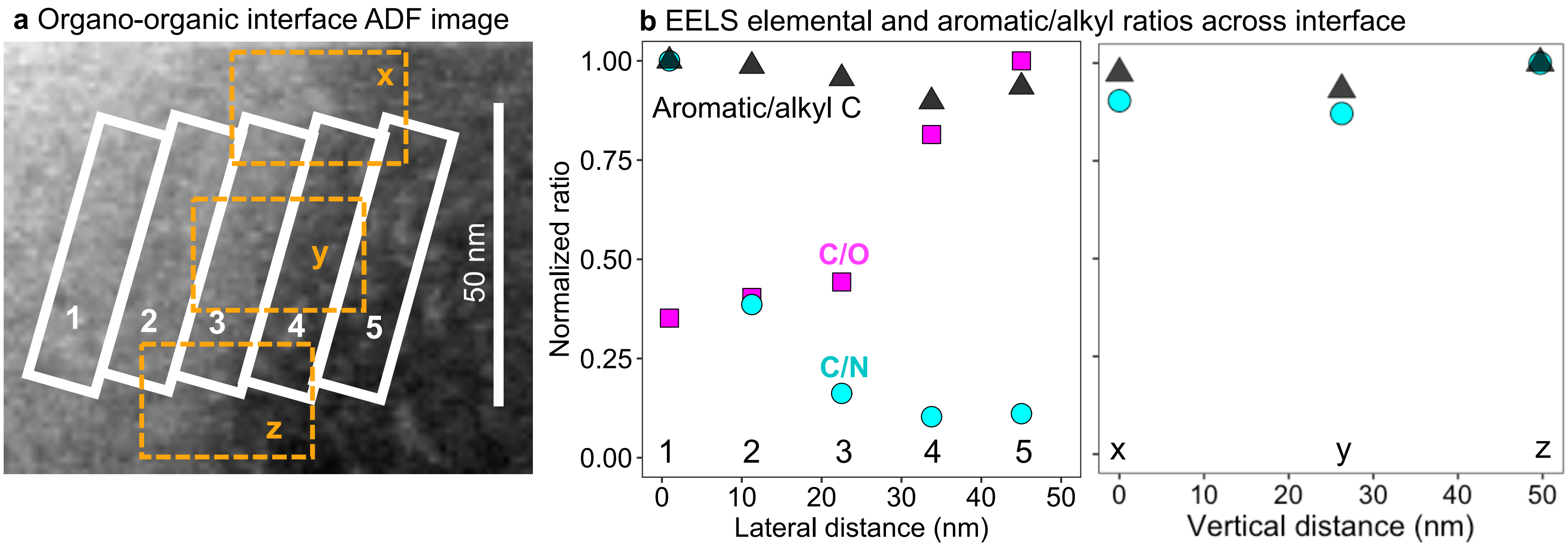

Supplementary Figure 4**. Electron energy loss spectroscopy vertical boxes along interface. a** Annular dark field (ADF) STEM detail of the interface between organic phases in a soil thin section. Boxes 1 through 5 indicate regions used to compute average electron energy loss (EEL) spectra (~540 individual spectra per box) across the interface (lateral distance), and boxes x through z indicate regions to assess variability along the interface (~620 individual spectra per box) (vertical distance). **b** Normalized (maximum=1) C/N, C/O and aromatic/alkyl C across the interface (lateral distance), and C/N and aromatic/alkyl C along the interface (vertical distance).

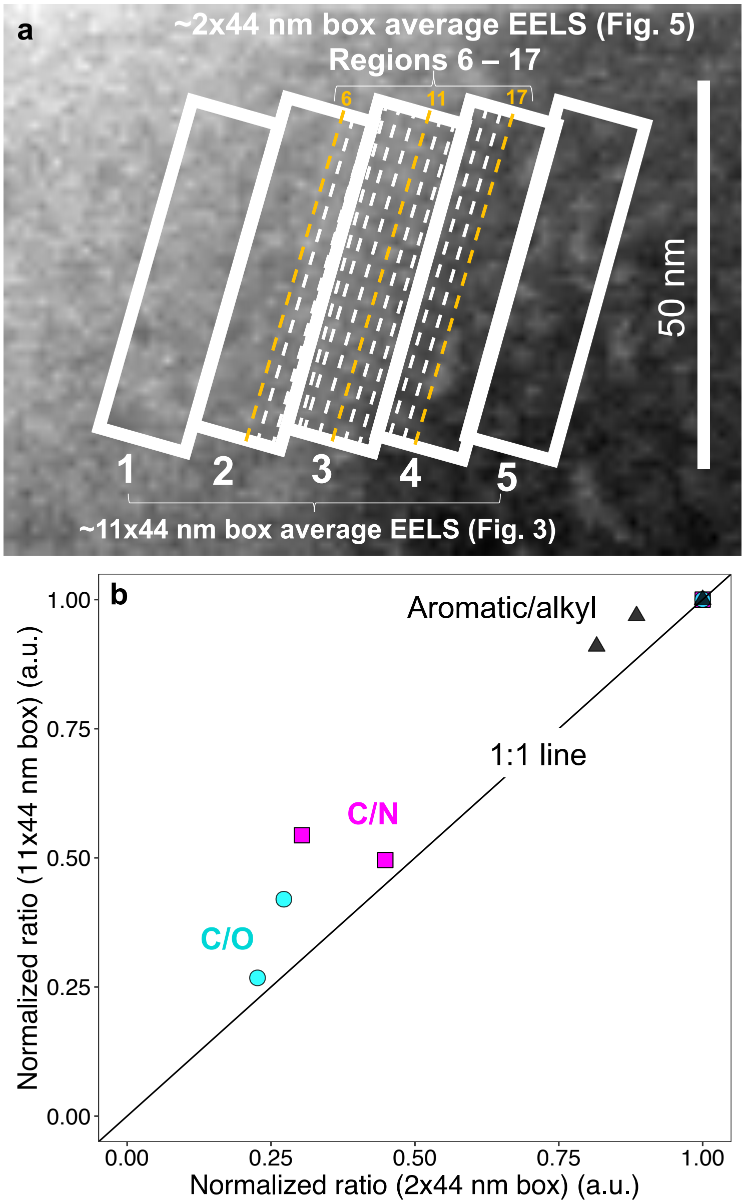

Supplementary Figure 5**. Relationship between 11x44 and 2x44 nm box average electron energy loss spectroscopy measurements.** Annular dark field (ADF) image of electron energy loss spectroscopy (EELS) analysis regions across organo-organic interface. **b** Relationship between measurements made using 2x44 nm box analysis regions (locations 6, 11, and 17) at the approximate center of 11x44 nm box analysis regions (2, 3, and 4) across organo-organic interface (main text Fig. 5). Points represent normalized (maximum = 1) calculated ratios for average spectra (~540 individual spectra for 11x44 nm boxes and ~90 spectra for 2x44 nm boxes).

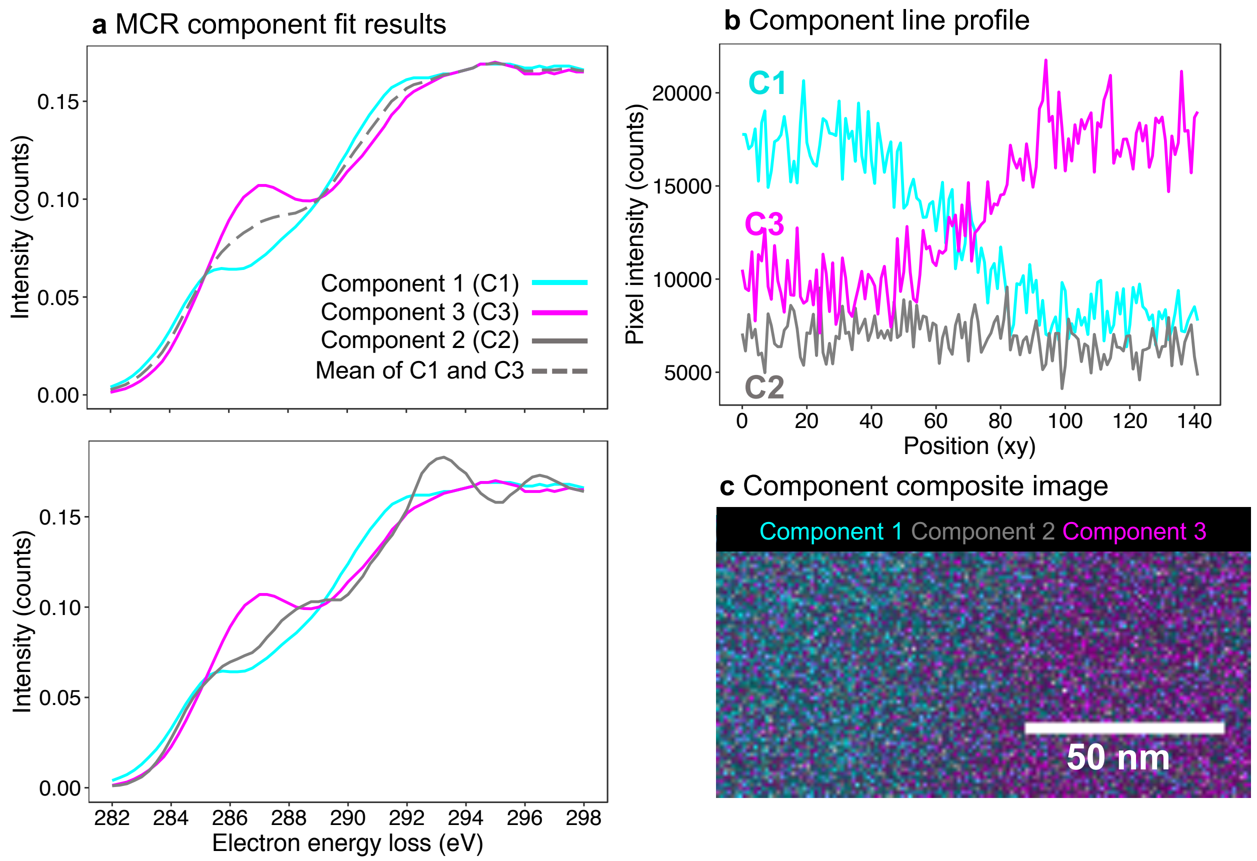

Supplementary Figure 6**. Three-component multivariate curve resolution (MCR) fit results**. **a** Plot of components 1 (C1), 2 (C2), and 3 (C3), and the arithmetic mean of C1 and C3 (dotted line). C1 and C3 correspond to components shown in main text **Fig. 3**. Component 2 (C2) is similar to the mean of C1 and C3, with additional noise compared to the arithmetic mean. **b** Component distribution across the analysis area. Component 2 does not vary appreciably across the sample. **c** Three-component composite image.

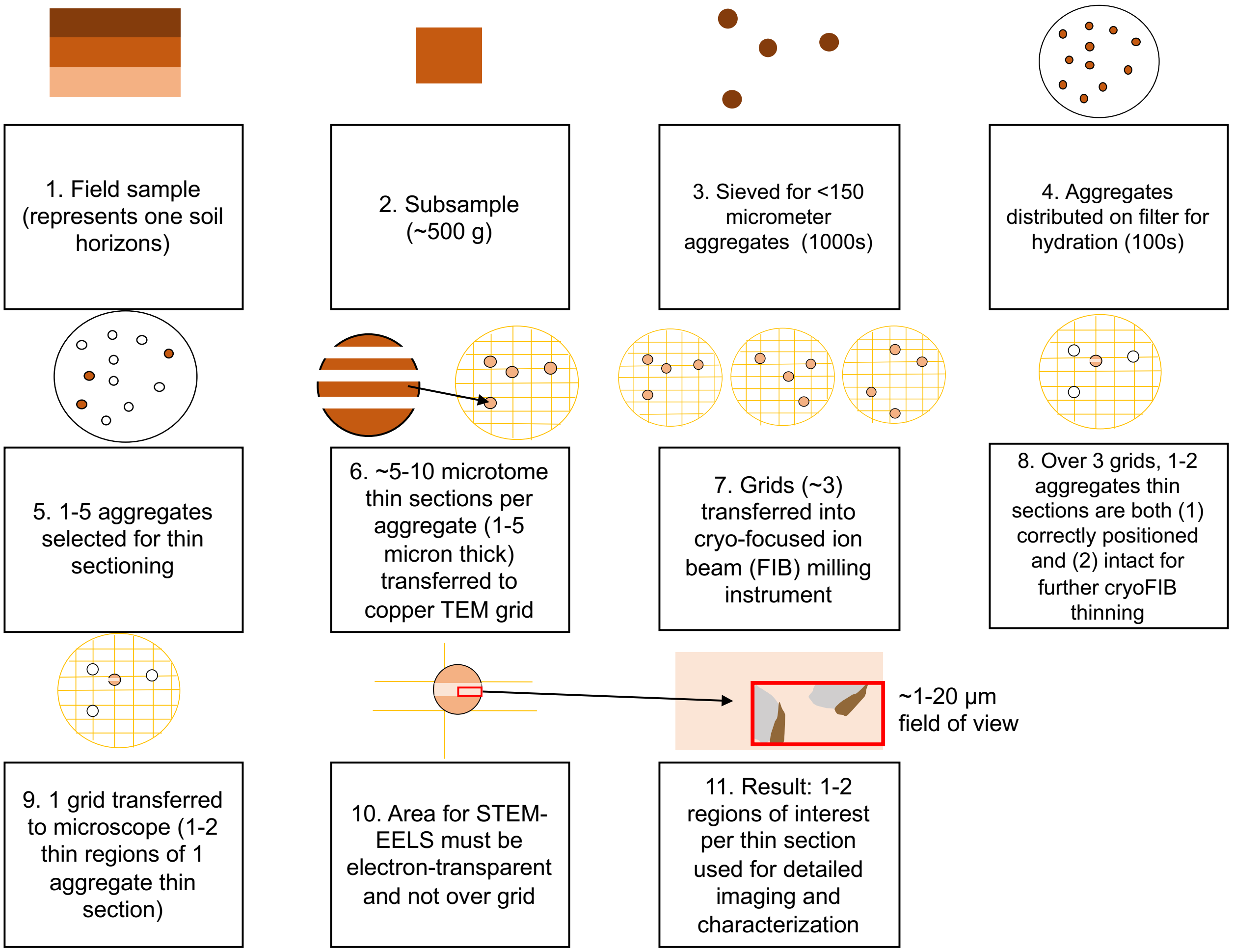

Supplementary Figure 7**. Analysis pipeline.** Analysis pipeline for preparation and analysis of electron-transparent regions of soil microaggregate thin-sections using scanning transmission electron microscopy (STEM) with electron energy loss spectroscopy (EELS).

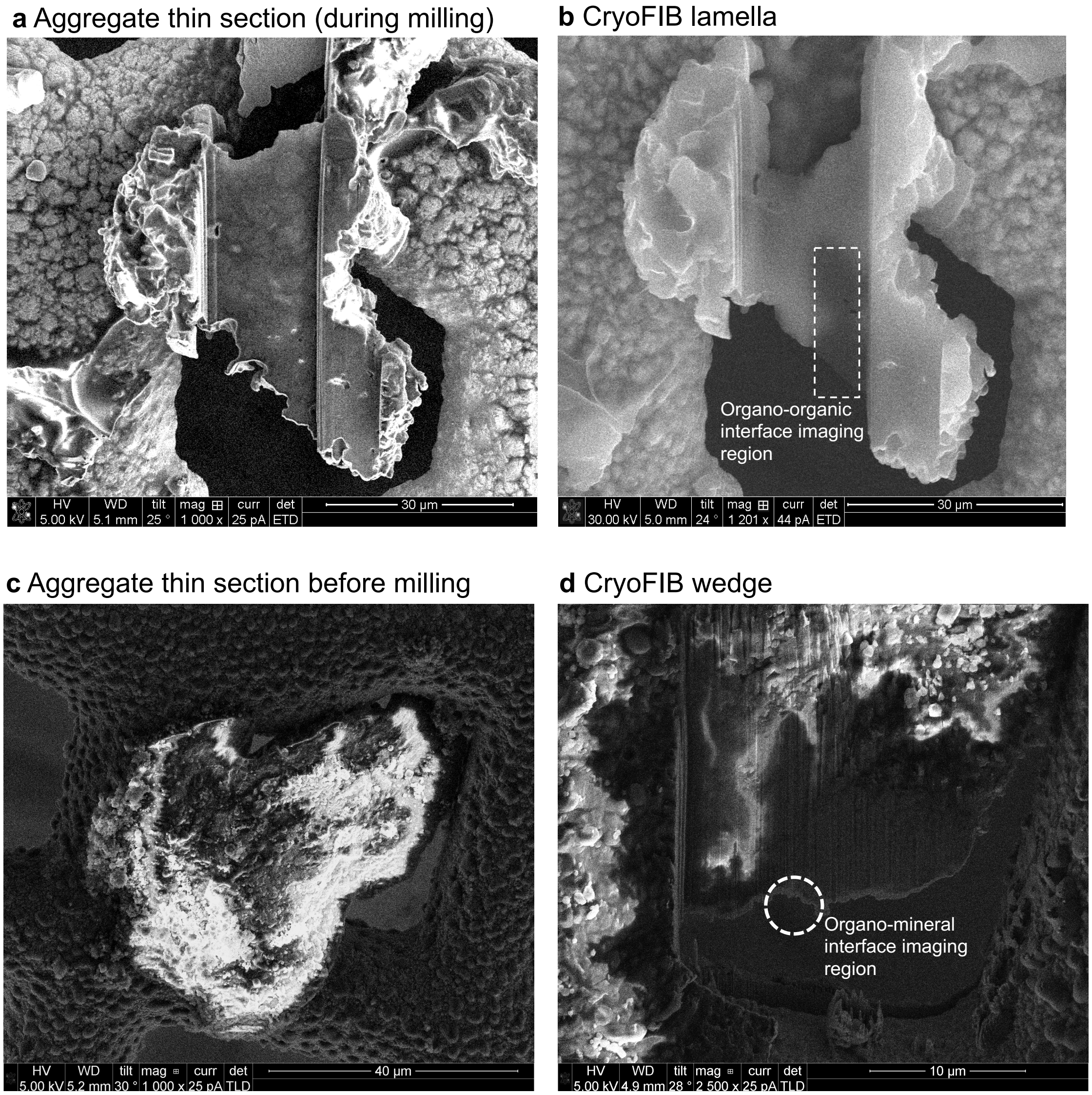

Supplementary Figure 8**. Sample preparation.** Cryogenic focused ion beam (FIB) sample preparation for scanning transmission electron microscopy-electron energy loss spectroscopy (STEM-EELS) measurements across interfaces of organo-organic and organo-mineral phases in volcanic soil samples. **a** Cryo-ultramicrotomed thin section of sample used for organo-organic interface analysis on grid, with initial additional thinning by cryo-FIB. Image contrast is increased to show differences associated with mineral components across the sample. **b** Cryo-FIB thinned lamella, with electron-transparent area for STEM-EELS imaging. The white rectangle indicates the approximate area of high-spatial resolution imaging. **c** Initial cryo-ultramicrotomed thin section for organo-mineral interface analysis on grid. **d** Cryo-FIB thinned wedge, with electron-transparent edge for STEM-EELS imaging. The white circle indicates the approximate area of high-spatial resolution imaging.

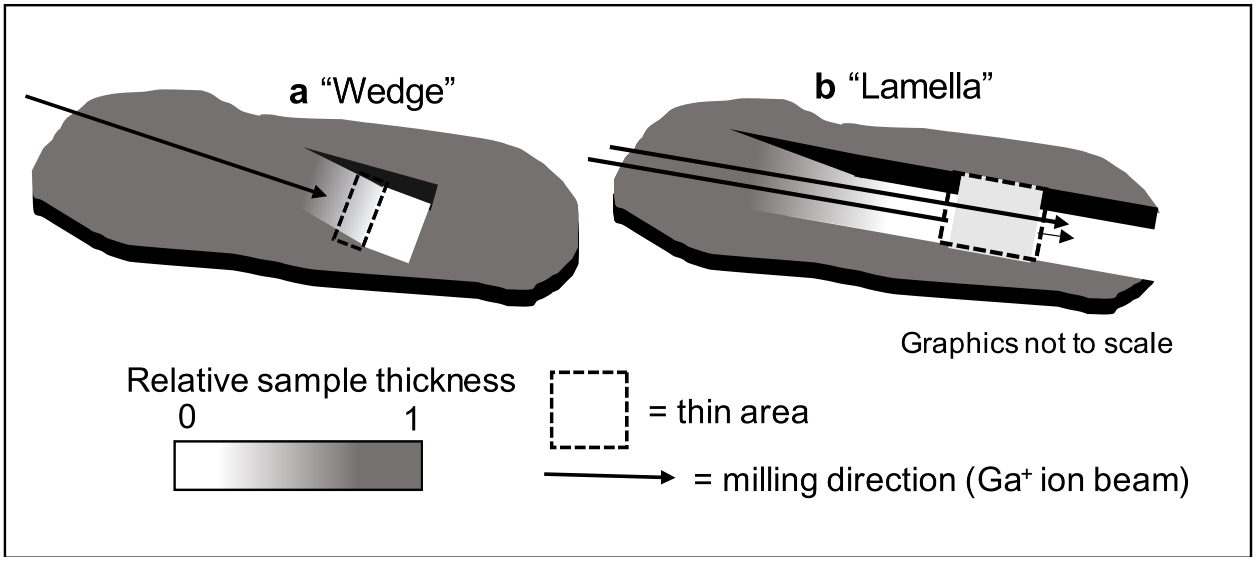

Supplementary Figure 9. **Simplified schematic of milling approaches. a** Milling to create a wedge with a gradient of thickness results in a thin edge for imaging (Supplementary Fig. 7d). A remaining challenge is relatively small area of electron-transparent sample, and the lack of smoothing from below, which can create uneven sample topography. **b** Milling from above and below to create a lamella of thin material (Supplementary Fig. 7b) results in more electron-transparent sample of even thickness available for imaging, but the thin lamella may be less mechanically robust.

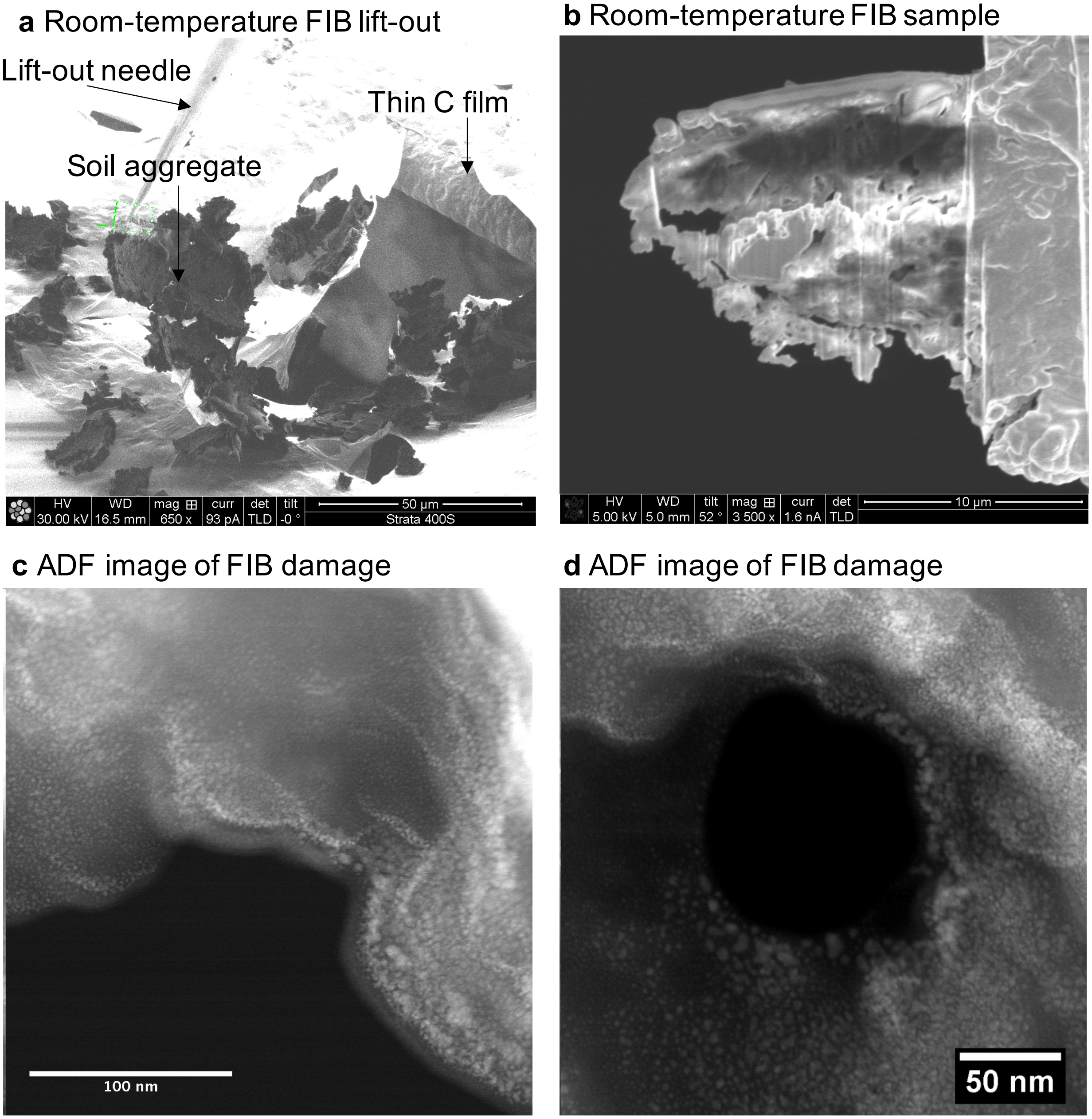

Supplementary Figure 10. **Room-temperature damage assessment**. Evidence of redeposition damage resulting from room temperature focused ion beam (FIB) sample preparation of a volcanic soil sample. **a** Scanning electron microscope (SEM) image within FIB instrument of soil aggregate thin section damage (fracturing and dispersal) from air-drying. FIB lift-out needle and attachment to broken section also shown. **b** SEM image showing evidence of curtaining (vertical striations) in soil thin section after lift-out and FIB thinning. **c, d** Annular dark field scanning transmission electron microscopy (ADF STEM) images collected of wavy distortion of sample structure, with irregular spots likely resulting from redeposition and/or gallium (Ga^{+}) implantation.

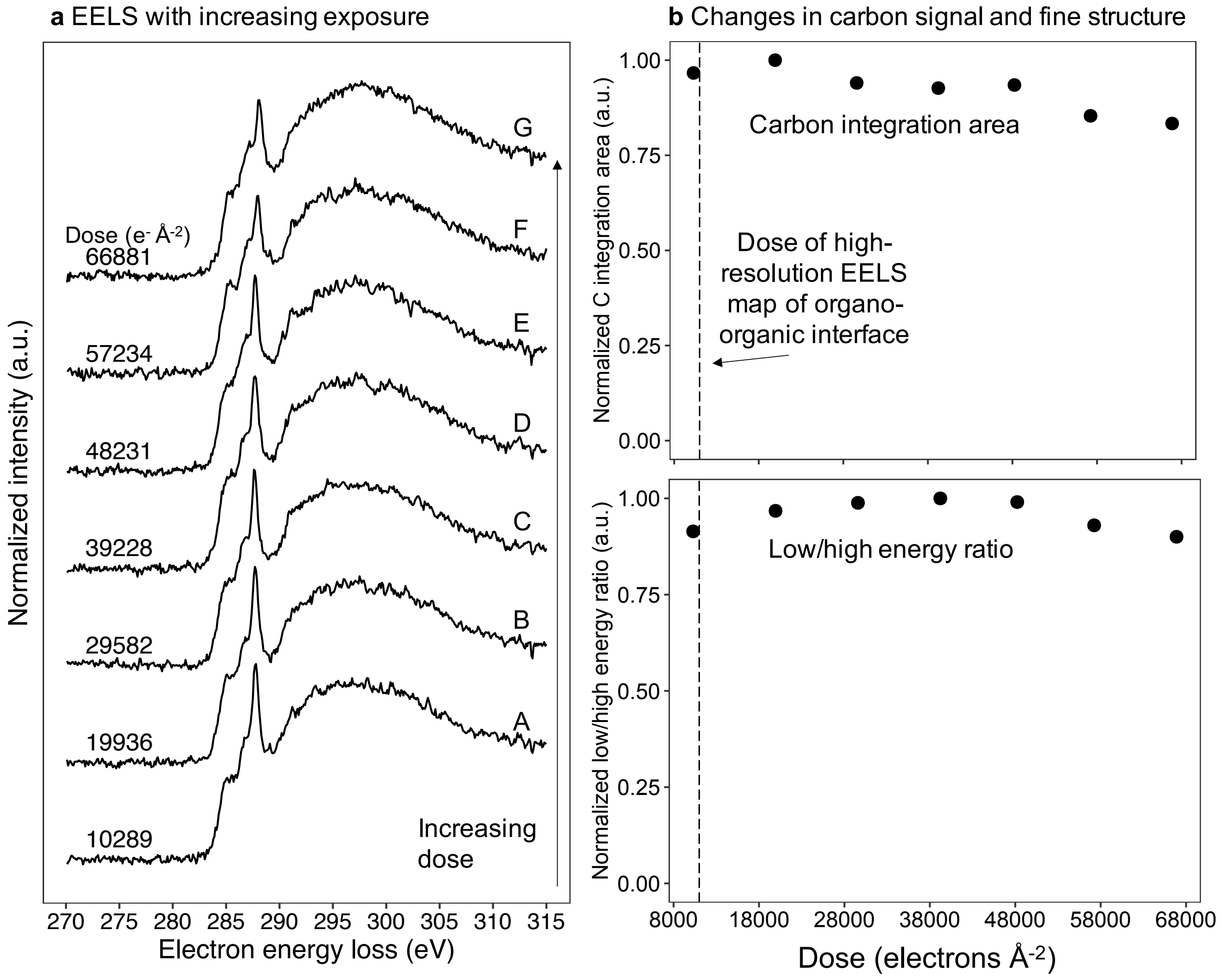

Supplementary Figure 11. **Damage test**. Repeated carbon (C) K-edge electron energy loss spectroscopy (EELS) measurements of a reference organo-mineral complex sample. **a** Normalized EEL spectra for doses increasing from ~10,000 - ~67,000 electrons (e^-) Å^{-2}. Each spectrum represents an average EELS measurement from repeated scans over an ~125x125 nm field of view. **b** Effect of electron dose on total C signal (integration from 280.0-315.0 eV) (for each spectrum in **a**). Dose applied to the high-resolution EELS map of an organo-organic interface (main text **Figs 3-5**) is indicated by the dashed vertical line. This panel also shows effect of electron dose on ratio of lower-energy (integrated 284.5-286.5 eV area) to higher-energy (integrated 286.5-289.0 eV area) features relative to total C integrated area.

Supplementary References

1. Inagaki, T. M. et al. Subsoil organo-mineral associations under contrasting climate conditions. *Geochim. Cosmochim. Acta* **270**, 244–263 (2020).

2. Hemingway, J. D. et al. Mineral protection regulates long-term global preservation of organic carbon. *Nature* **570**, 228-231 (2019).

3. Hemingway, J. D. et al. Assessing the blank carbon contribution, isotope mass balance, and kinetic isotope fractionation of the ramped pyrolysis/oxidation instrument at NOSAMS. *Radiocarbon* **59**, 179-193 (2017).

4. Hemingway, J. D. Ramped Pyrolysis/Oxidation (RPO) Database (Version 0.0.1) Data set. *Zenodo* http://doi.org/10.5281/zenodo.1158742 (2018).
